# Differential roles of CTP synthetases CTPS1 and CTPS2 in cell proliferation

**DOI:** 10.26508/lsa.202302066

**Published:** 2023-06-22

**Authors:** Norbert Minet, Anne-Claire Boschat, Rebecca Lane, David Laughton, Philip Beer, Hélène Asnagli, Claire Soudais, Tim Bourne, Alain Fischer, Emmanuel Martin, Sylvain Latour

**Affiliations:** 1 Laboratory of Lymphocyte Activation and Susceptibility to EBV Infection, Inserm UMR 1163, Imagine Institute, Paris, France; 2 Université de Paris, Paris, France; 3 Plateforme Spectrométrie de masse, Institut Imagine, Paris, France; 4 Laboratoire de Biochimie Métabolomique et Protéomique, Hôpital Necker Enfants-Malades, Assistance Publique-Hôpitaux de Paris (AP-HP), Paris, France; 5 Sygnature Discovery, Nottingham, UK; 6 Step-Pharma, Saint-Genis-Pouilly, France; 7 Collège de France, Paris, France; 8 Imagine Institute, Paris, France

## Abstract

The CTP nucleotide is a key precursor of nucleic acids metabolism essential for DNA replication. De novo CTP production relies on CTP synthetases 1 and 2 (CTPS1 and CTPS2) that catalyze the conversion of UTP into CTP. CTP synthetase activity is high in proliferating cells including cancer cells; however, the respective roles of CTPS1 and CTPS2 in cell proliferation are not known. By inactivation of *CTPS1* and/or *CTPS2* and complementation experiments, we showed that both CTPS1 and CTPS2 are differentially required for cell proliferation. CTPS1 was more efficient in promoting proliferation than CTPS2, in association with a higher intrinsic enzymatic activity that was more resistant to inhibition by 3-deaza-uridine, an UTP analog. The contribution of CTPS2 to cell proliferation was modest when CTPS1 was expressed but essential in absence of CTPS1. Public databases analysis of more than 1,000 inactivated cancer cell lines for CTPS1 or CTPS2 confirmed that cell growth is highly dependent of CTPS1 but less or not of CTPS2. Therefore, our results demonstrate that CTPS1 is the main contributor to cell proliferation.

## Abstract (precis)

The CTP nucleotide is essential for cell proliferation. This study explores the respective contributions of CTP synthetases 1 and 2 in cell proliferation. Compared with CTPS2, CTPS1 is the main contributor in association with its higher enzymatic activity.

## Introduction

As the building blocks of RNA and DNA, and as substrates of various cellular processes, nucleotides are the key to most normal and pathological cellular and metabolic processes. Nucleotide levels are therefore tightly regulated, including through de novo synthesis, recycling, and/or salvage pathways. The pyrimidine nucleotide CTP is known to be in limiting concentrations in cells, in contrast to the other “main” nucleotides (ATP, GTP, TTP, and UTP). CTP has also been linked to the synthesis of phospholipids and the sialylation of proteins (1, 2, 3). Furthermore, it was recently shown that the limited availability of CTP shapes viral evolution (4), and CTP is a substrate for viperin, an enzyme that catalyzes the formation of ddhCTP, an antiviral nucleotide (5).

CTP arises from two sources, one dependent on the nucleotide salvage pathway, whereas the second involves de novo synthesis (6). The salvage pathway recycles the nucleoside cytidine, a product of nucleic acid degradation. Cytidine is transformed into CTP via the synthesis of CMP and CDP. The de novo synthesis pathway of CTP is dependent on the enzymatic activity of CTP synthetases (CTPS). The CTPS activity is a two-step reaction involving two distinct enzymatic activities, a kinase and a glutaminase activity. CTPS catalyzes the ATP-dependent amination of UTP into CTP using ammonia (NH_3_) transferred from the parallel hydrolysis of glutamine. In humans, CTPS activity is dependent on two enzymes with highly conserved structural identity; the CTP synthases 1 and 2 (CTPS1 and CTPS2) that are, respectively, encoded by the *CTPS1* and *CTPS2* genes. CTPS1 and CTPS2 share more than 75% of identity and are relatively well-conserved through species (7). CTPS1 and CTPS2 contain two enzymatic domains, a synthetase/kinase domain and a glutaminase domain separated by a linker region. The C-terminal part is a regulatory domain with several sites of phosphorylation and is the most variable region between CTPS1 and CTPS2. CTPS1 is regulated by phosphorylation (8, 9, 10), ubiquitination (11), and polymerization (12, 13). CTPS1 forms dimers and tetramers, representing inactive and active forms of the enzyme, respectively. Moreover, tetramers of CTPS1 can polymerize into higher order structures known as filaments, rod and rings, or *cytoophidia*, whose function remains debated (14, 15, 16, 17). These different steps of CTPS1 containing supramolecular structure are influenced by the availability of substrates and products (12, 16, 18, 19). Regarding the regulation of CTPS2, little is known. Two studies have reported that CTPS2 shares with CTPS1 at least some of these regulatory mechanisms, including phosphorylation and filament formation (20, 21).

Both CTPS1 and CTPS2 proteins are expressed in all tissues (www.biogps.org; www.proteinatlas.org). CTP synthase activity is considered to be low in normal tissues whereas higher in proliferating tissues such as tumor cells, likely allowing malignant cells to overcome the CTP concentration bottleneck (22, 23, 24). However, limited information is currently available on the respective role of CTPS1 and CTPS2 in proliferation. Notably, evidence of the roles of CTPS1/2 in proliferation was obtained by indirect approaches using inhibitors of CTPS activity (25, 26). Furthermore, these inhibitors have limited specificity with side effects on other metabolic pathways (27). To date, the first and only direct evidence of the role of CTPS activity in cell proliferation has been provided by the recent identification of a cohort of immunodeficient patients harbouring a deleterious homozygous mutation in CTPS1 that results in a strongly reduced CTPS1 expression and activity (28, 29). CTPS1 expression was found to be up-regulated in T lymphocytes following stimulation through the T cell receptor for antigen (TCR) and necessary for their expansion during antigen-specific immune response. Importantly, CTPS1 was shown to be selectively required for the proliferation of activated T lymphocytes but not for their differentiation in effector cells.

To better understand the role respective of CTPS1 and CTPS2, we examined the impact of *CTPS1* and/or *CTPS2* gene inactivation by CRISPR–Cas9 on cell proliferation, viability, and overall CTPS activity in two cell models and using recombinant proteins. We show that *CTPS1* and *CTPS2* are partially redundant and not equivalent to promote cell proliferation in correlation with differences in their enzymatic activity. Analysis of public databases of more than 1,000 inactivated cancer cell lines for *CTPS1* or *CTPS2* confirmed that cell growth is highly dependent on CTPS1 but not or less on CTPS2. In conclusion, our study documents that CTPS1 and CTPS2 are critical factors of cell proliferation with partially redundant roles.

## Results

### CTPS1 and CTPS2 expression in different cell lines

**Figure 1.**
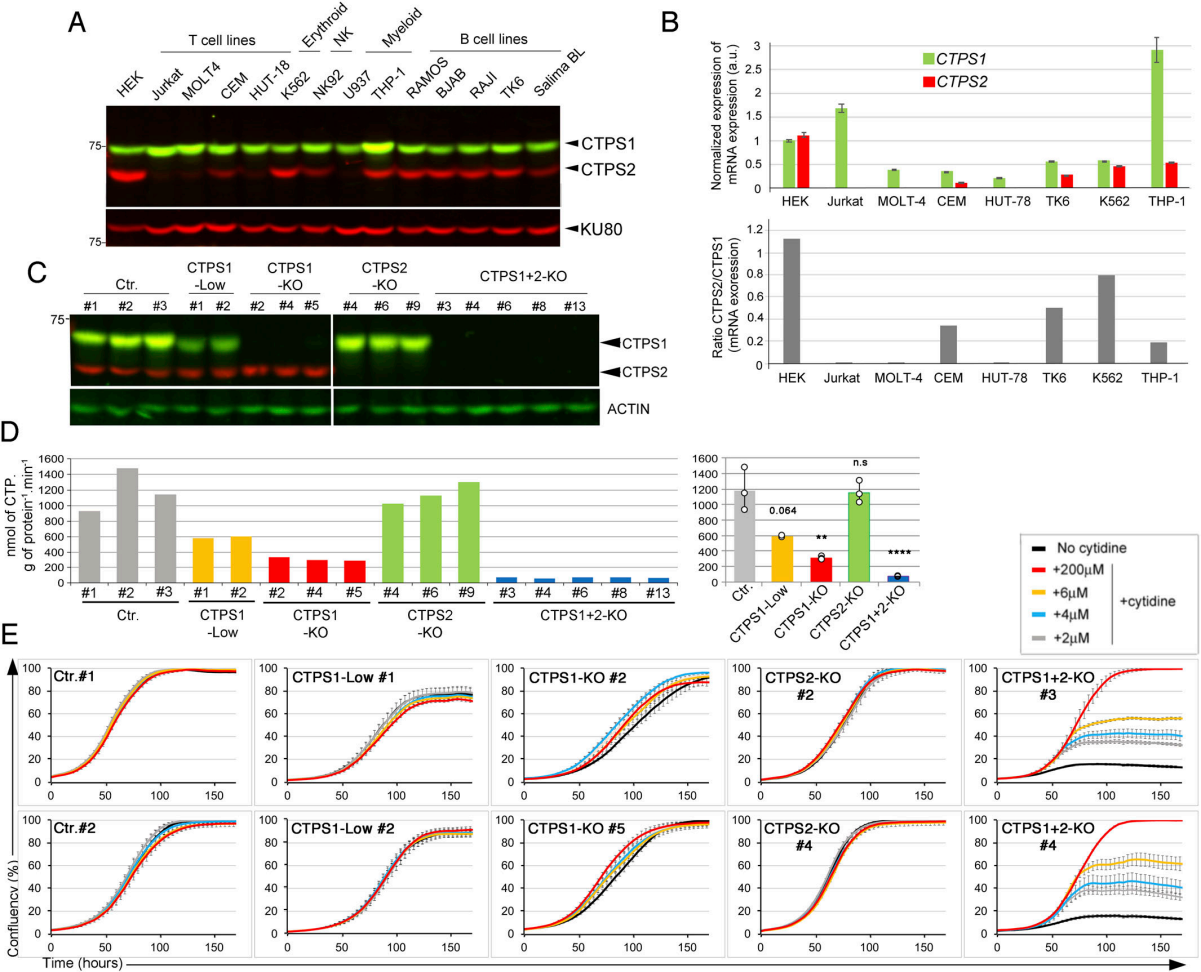
Differential roles of CTPS1 and CTPS2 in proliferation of HEK cells. **(A, B)** Expression of CTPS1 and CTPS2 in different cell lines. **(A)** Western blots of total cell lysates showing CTPS1 and CTPS2 expression in different hematopoietic cell lines with the exception of HEK, that is, a non-hematopoietic cell line. KU80 as the loading control. The weaker band corresponding to CTPS2 detected in Jurkat, MOLT-4, HUT-78 and U937 is non-specific. **(B)** Expression of *CTPS1* and *CTPS2* mRNA normalized to *GAPDH* mRNA by qRT-PCR (upper panel). Ratio of *CTPS2*/*CTPS1* mRNA expression from qRT-PCR data (lower panel). **(C)** Western blots showing the expression of CTPS1 and CTPS2 in HEK cell lines derived from the sub-cloning of polyclonal bulk cultures in which *CTPS1* (CTPS1-KO or CTPS1-low) or *CTPS2* (CTPS2-KO) have been inactivated by CRISPR–Cas9 genome editing. Double CTPS1- and CTPS2-inactivated cell lines (CTPS1+2-KO) were obtained from the CTPS1–KO#5 clone. Two wild-type cell lines obtained from the sub-cloning of cells targeted by CRISPR for *CTPS1* (Ctr.#2 and #3) or control HEK cells (Ctr.#1) are also shown. **(D)** CTPS activity measured from cell lysates of control (Ctr.) or CTPS1- and/or CTPS2-deficient HEK cells (CTPS1-KO, CTPS1-low, CTPS2-KO, and CTPS1+2-KO). All cells were maintained without cytidine supplementation before the activity measurement, excepted for the CTPS1- and CTPS2-KO cells which were expanded in the presence of 200 µM of cytidine, washed, and cytidine-starved for 48 h before CTPS activity was measured. Means with SEM of CTPS activity values for each group of clones are shown in the right panel. Two-tailed unpaired *t* tests against Ctr. values; n.s., no significance; **P < 0.01; ****P < 0.0001. **(E)** Confluency curves as percentages (%) showing the proliferation of control (Ctr.) or CTPS1- and/or CTPS2-deficient HEK cells (CTPS1-KO, CTPS1-low, CTPS2-KO, and CTPS1+2-KO). Confluency measurement using an IncuCyte Zoom system. Cells were seeded for 24 h, and then treated with the indicated concentrations of cytidine. **(A, B, C, D)** Data of one representative experiment of two (C, D) or three (A, B) independent experiments. **(E)** Data of one representative experiment of three independent experiments.

We examined the levels of the CTPS1 and CTPS2 proteins in a variety of cell lines of hematopoietic origin and in the non-hematopoietic embryonic kidney cell line HEK 293T (hereafter designated as HEK) by Western blot and qRT-PCR (Fig 1). CTPS1 was found to be expressed at protein level in all tested cell lines, whereas CTPS2 expression was variable depending on cell origin. CTPS2 was not detectable or weakly expressed in some cancer cell lines of T lymphoid origin like MOLT-4 (a human T lymphoblast line from an acute lymphoblastic leukemia), HUT-78 (derived from cutaneous T lymphocytes from a patient with Sezary syndrome), and Jurkat (an acute T-cell leukemia) cells, whereas it was detectable in CCRF–CEM cells, an acute lymphoblastic T-cell leukemia/T-ALL (Fig 1A). CTPS2 was not detectable in the U937 cell line of myeloid origin. All B lymphoblastoid cell lines expressed CTPS2 as well as the NK92, K562, and THP-1 cell lines of NK, erythroid, and myeloid origin, respectively. HEK cells expressed both CTPS1 and CTPS2. Expression of CTPS2 was higher in HEK than in cell lines of hematopoietic origin. Levels of *CTPS1* and *CTPS2* transcripts analysed by qRT-PCR in several of these cell lines were consistent with protein expression (Fig 1B). Notably, HEK cells exhibited the highest level of *CTPS2* transcripts and ratio of *CTPS2*/*CTPS1* mRNA. To determine the respective roles of CTPS1 and CTPS2 in cell proliferation, we thus decided to inactivate *CTPS1* and/or *CTPS2* by CRISPR–Cas9 genome editing in HEK cells (that express high levels of both CTPS1 and CTPS2) and Jurkat cells (that do not express CTPS2). Given the key role of CTPS1 in T lymphocytes (28, 29), Jurkat cells represented a valid T-cell malignancy model to selectively study the role of CTPS1. Furthermore, both cell lines (HEK and Jurkat) have been extensively used and characterized in the past and are easy to manipulate and to transfect/infect.

### Inactivation of CTPS1 and/or CTPS2 in HEK cells

Single guide RNAs (sgRNA) targeting sequences in exons 6 and 10 of *CTPS1* or 5 and 10 of *CTPS2* were first introduced into HEK cells by transfection using a plasmid containing the sgRNA and sequences for Cas9 and puromycin resistance genes. After puromycin selection, cells were maintained in culture in the presence of cytidine to provide intracellular CTP to the cells through the salvage pathway to avoid counterselection of CTPS1- or CTPS2-deficient cells. Cytidine is a substrate of the salvage pathway, and supplementation with cytidine indeed bypasses the requirement of CTPS activity for CTP synthesis (30). Bulk cultures were first analysed by Western blot to identify the most efficient guides (data not shown), then sub-cloned and clones analysed for CTPS1 and/or CTPS2 expression by Western blot (Fig 1C). Several clones either not expressing CTPS1 or CTPS2 (CTPS1- KO or CTPS2-KO) or expressing decreased levels (∼50%) of CTPS1 (CTPS1-low) were selected for further studies. Clones deficient for both CTPS1 and CTPS2 (CTPS1+2-null) were obtained from a CTPS1-deficient clone that was transfected with a CRISPR–Cas9 vector containing guides targeting *CTPS2*. We first analysed the CTPS enzymatic activity in cell lysates of CTPS1- and/or CTPS2-deficient cells. Although the absence of CTPS2 had no significant impact on the global level of CTPS activity (Fig 1D), cells expressing decreased amounts of CTPS1 showed a strongly reduced CTPS activity (∼50%) compared with controls. In cells in which CTPS1 expression was completely abrogated, CTPS activity levels were further decreased to 20% of the CTPS activity of control wild-type cells. The combined absence of CTPS1 and CTPS2 (in CTPS1+2 null cells) as it could be expected almost completely abolished the CTPS activity. These data suggest that the contribution of CTPS2 to the total CTPS activity of HEK cells is low (10–20%) and negligible when CTPS1 is expressed.

**Figure S1.**
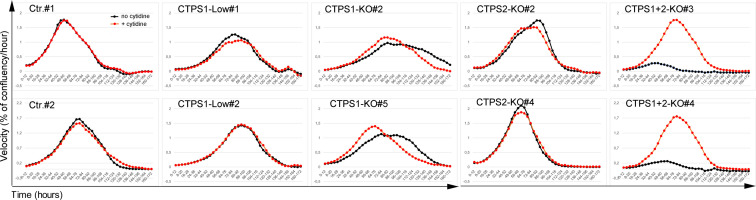
Different contributions of CTPS1 and CTPS2 to proliferation of HEK cells. Proliferation velocity, corresponding to Fig 1E. Velocity for each population in presence (red dotted line) or absence (black dotted line) of cytidine supplementation (200 μM), corresponding to the increase in confluency per hour on 12-h periods. Data of one representative experiment of three independent experiments.

**Figure S2.**
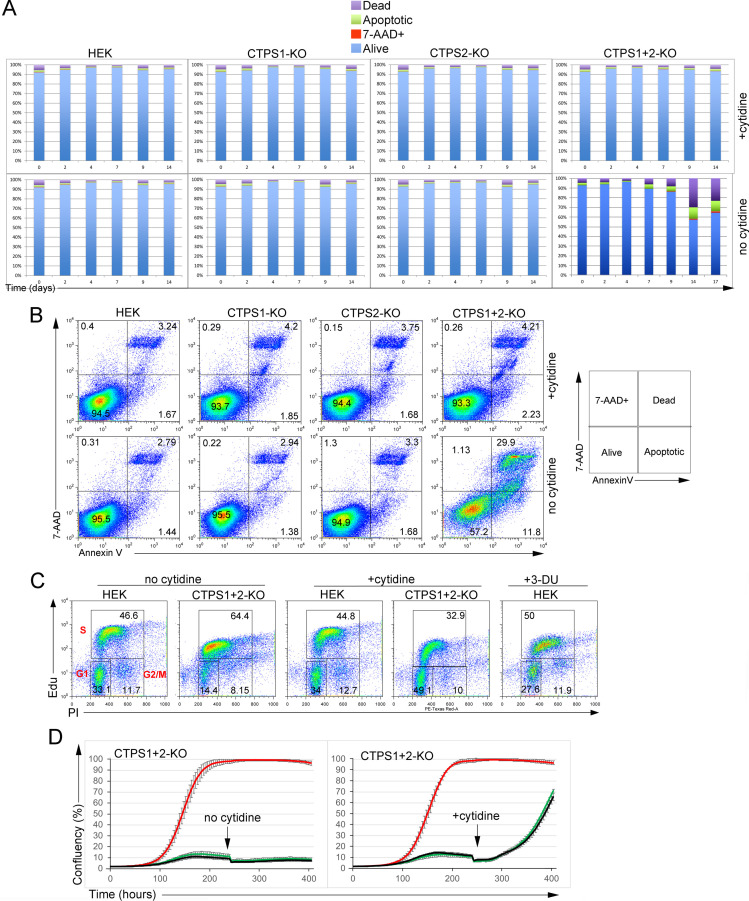
Inactivation of CTPS1 and CTPS2 in HEK cells induces abnormal cell cycle with a moderate level of cell death. **(A, B)** Analysis of apoptotic/cell death in cell-cultures of CTPS1- and/or CTPS2-deficient HEK cell lines (CTPS1-KO, CTPS2-KO, and CTPS1+2-KO). Cells were seeded for 24 h, and then cultured in the presence or absence of 200 M cytidine. Annexin V and 7-AAD apoptotic/cell death markers were analysed by flow cytometry at day 2, 4, 7, 9, 14, and 17 of culture. **(A, B)** Bar graphs from flow cytometry data presented in (B) showing the percentage (%) of dead, 7-AAD+, alive, and apoptotic cells. **(B)** FACS dot-plots of annexin V and 7-AAD staining at day 14. On the right, correspondence between staining gates and cell phenotypes. **(C)** FACS dot-plots of cell cycle analysis showing staining of EdU and PI incorporation in control HEK or and CTPS1+2-KO cells treated or not with 200 μM cytidine or 40 μM 3-deaza-uridine (3-DU) as indicated. Gates corresponding to G1, G2/M, and S phases of the cell cycle are indicated in red in the first left dot-plot. **(D)** Confluency curves in percentages (%) showing the proliferation of HEK cells deficient for both CTPS1 and CTPS2 (CTPS1+CTPS2-KO). Confluency measurement by IncuCyte Zoom system. Cells were seeded for 24 h, and then cultured in the presence or absence (black line) of 200 μM of cytidine (red line) or 40 μM of 3-deaza-uridine (3-DU) (green line). The black arrows indicate when cytidine was added. **(A, B, C)** Data of one representative experiment of three independent experiments.

**Figure S3.**
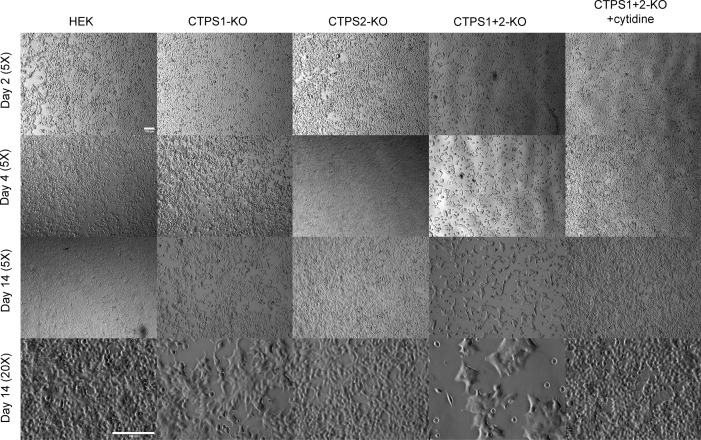
Inactivation of CTPS1 and CTPS2 in HEK cells induces an increase in cell size. Analysis of cell confluency of CTPS1- and/or CTPS2-deficient HEK cell lines (CTPS1-KO, CTPS2-KO and CTPS1+CTPS2-KO). Cells were seeded for 24 h, and then cultured in the presence or absence of 200 M cytidine. Pictures taken with a Zeiss Axio Vert A1 at day 2, 4, and 14, of culture. Data of one representative experiment of two independent experiments except that day 14 was only tested one time. Scales are depicted by horizontal white lines corresponding to 100 μM.

We next assessed the effect of *CTPS1* and/or *CTPS2* inactivation on the proliferation of HEK cells. Growth of control, CTPS1-KO, CTPS1-low, CTPS2-KO, and CTPS1+2-KO cells in the presence or absence of cytidine supplementation were evaluated using a live-cell analysis system that measures cell confluency over time. Addition of cytidine had no effect on the proliferation of control, CTPS2-deficient cells, or cells with reduced CTPS1 levels, supporting the notion that CTP production by these cell lines was sufficient to sustain cell growth (Fig 1E). Although CTPS1-deficient cells were able to proliferate in the absence of cytidine supplementation, their proliferation rate/velocity was diminished as cytidine supplementation increased their cell growth (Figs 1E and S1). These data indicate that the decreased CTPS activity observed in CTPS1-deficient cells impacted their ability to proliferate normally. In striking contrast, cells deficient for both CTPS1 and CTPS2 were unable to proliferate in the absence of cytidine supplementation. Cytidine supplementation restored their proliferation in a concentration-dependent manner (Fig 1E). In the absence of cytidine supplementation, non-proliferating CTPS1+2-KO cells persisted in the culture for at least up to 10 d, and only after 14 d without cytidine, were a few dead cells observed accumulating in the culture (Fig S2A and B). These cells displayed an abnormal S phase of the cell cycle characterized by a decrease of the EdU staining/incorporation and less cells in the G1 phase when compared with wild-type HEK cells (Fig S2C), and intriguingly, they exhibited an abnormally large size (Fig S3). This abnormal S phase is consistent with the absence of proliferation of CTPS1+2-KO cells that are in a quiescent state (defined as a reversible non-proliferating state). These cells were able to resume proliferation as soon as cytidine was added to the medium indicating their quiescent status (Fig S2C and D). Of note, the proportion of cells in G1 phase was increased again (like in wild-type HEK cells) indicating that there is an active transition from G1 phase to S phase in these cells as expected for cells that are proliferating (again).

**Figure 2.**
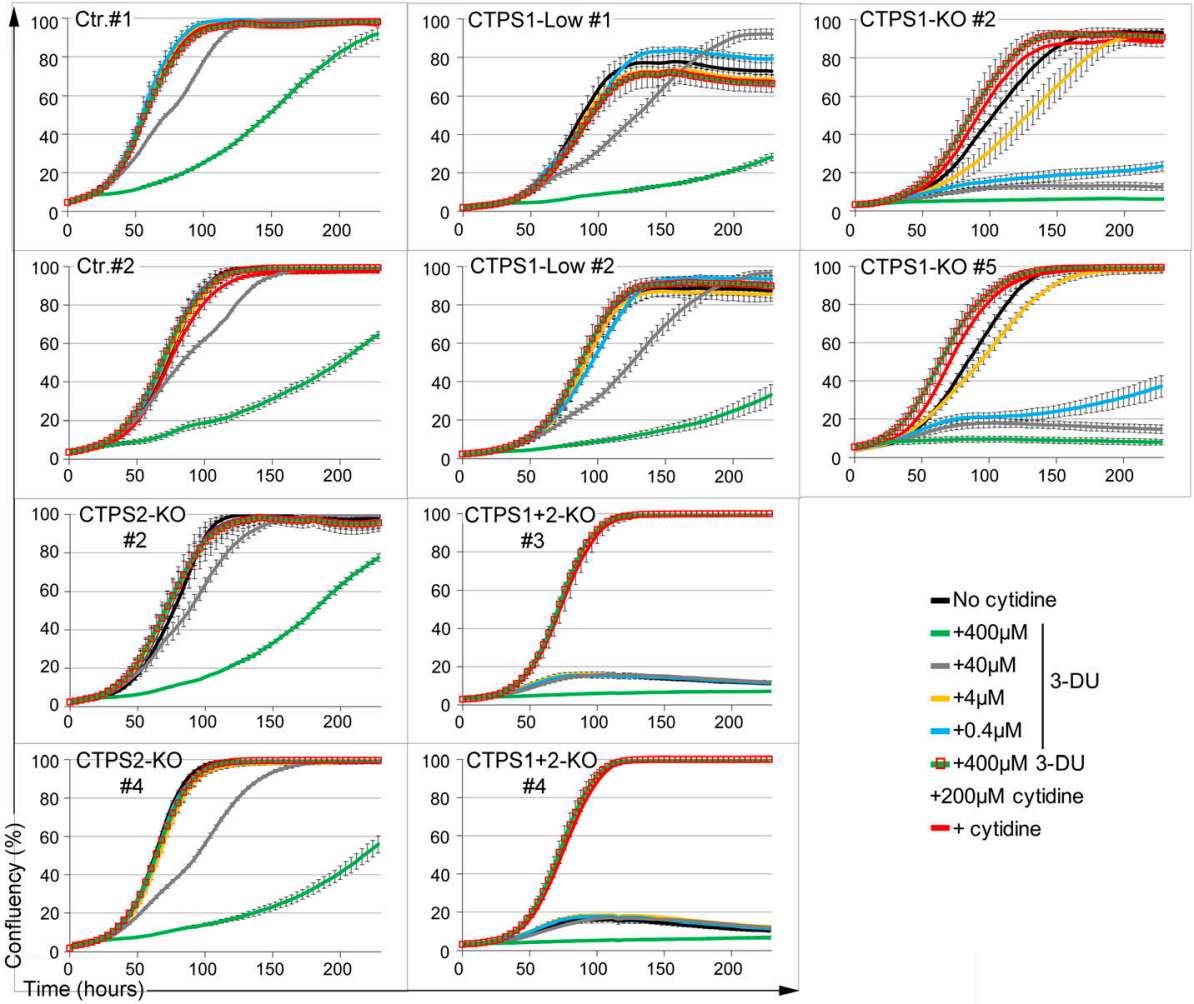
Response of CTPS1- and/or CTPS2-deficient HEK cells to 3-deaza-uridine. Confluency curves as percentages (%) showing the proliferation of control (Ctr.) or CTPS1- and/or CTPS2-deficient HEK cell lines (CTPS1-KO, CTPS1-low, CTPS2-KO, and CTPS1+2-KO). Confluency measurement using an IncuCyte Zoom system. Cells were seeded for 24 h, and then treated with the indicated concentrations of 3-deaza-uridine (3-DU) in the presence or absence of cytidine or cytidine alone at 200 µM. Data of one representative experiment of three independent experiments.

We then analysed the sensitivity of these different cell lines to 3-deaza-uridine (3-DU), a uridine analog known to be a selective inhibitor of CTPS activity, by competing with the UTP substrate (31, 32). Cells treated with different concentrations of 3-DU were evaluated for cell growth. First, we observed that the negative impact of 3-DU on proliferation was strictly due to selective inhibition of CTPS activity as supplementation of the cells with cytidine rescued cell proliferation to control levels (Fig 2). Control/wild-type and CTPS2-KO cells either responded weakly or not at all to low concentrations of 3-DU, whereas proliferation of CTPS1-low and CTPS1-KO cells in response to similar concentrations of 3-DU was notably reduced or completely abolished, respectively. These results indicate that 3-DU can be used to titrate CTPS activity and suggest that CTPS2 is more prone to 3-DU inhibition than CTPS1. This could also indicate that the CTPS2 activity is lower than that of CTPS1 as the result of a reduced affinity for UTP (compared with CTPS1) that is more easily counteracted/displaced by the 3-DU. This also confirms that CTPS1 is the main contributor to CTPS activity in HEK cells. Taken together, these data indicate that CTPS1 is an important factor for the proliferation of HEK cells, whereas the contribution of CTPS2 is rather modest in this model, although it appears to be essential when CTPS1 is absent (e.g., in CTPS1-KO cells).

### Inactivation of CTPS1 in Jurkat cells

**Figure 3.**
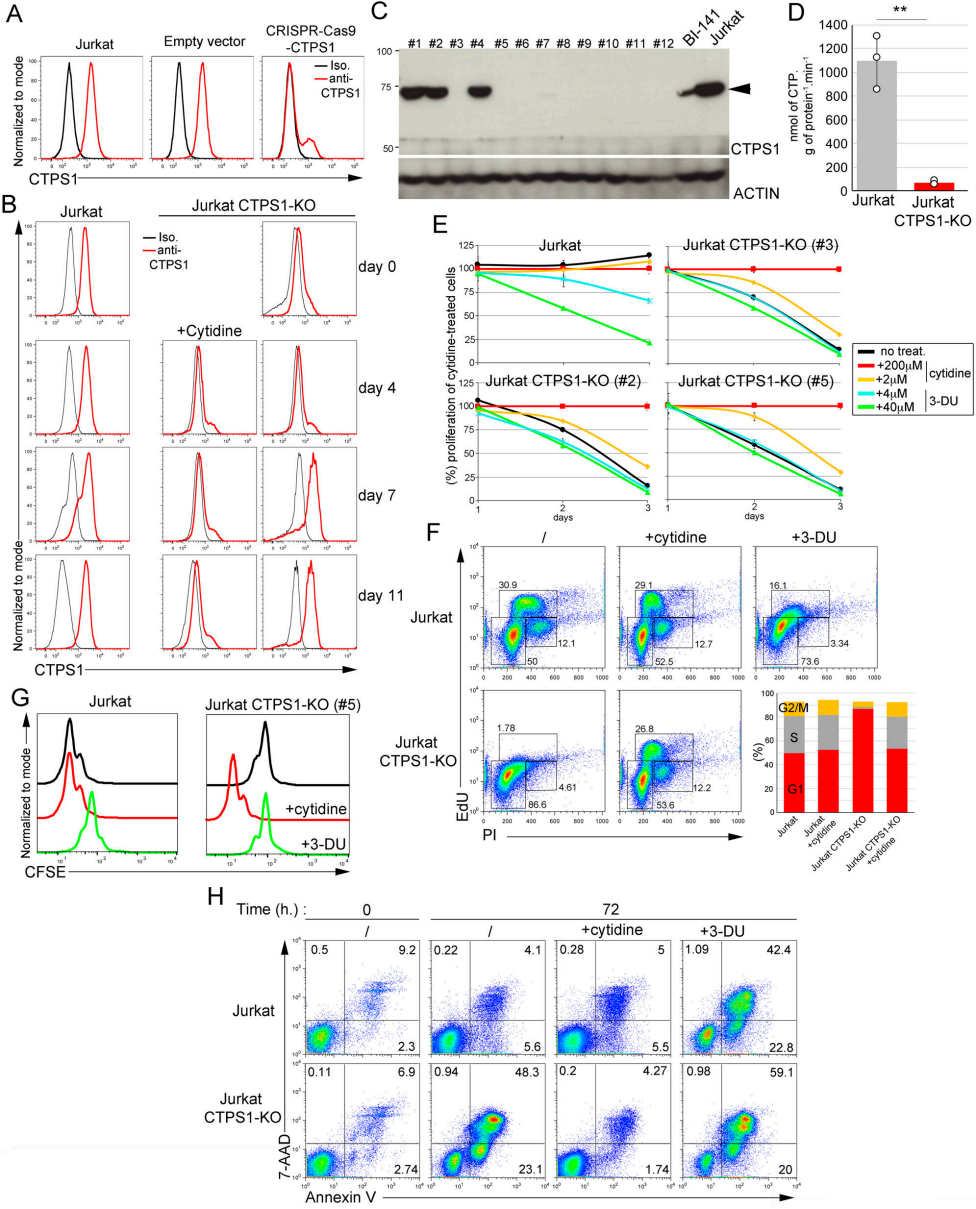
CTPS1 is required for the survival and proliferation of Jurkat cells. **(A)** Histograms from FACS analysis showing intracellular staining of CTPS1 in control Jurkat cells and Jurkat cells in which *CTPS1* has been targeted by CRISPR–Cas9 genome editing (CRISPR–Cas9–CTPS1) or with an empty vector which does not contain the guide (empty vector). Targeted cells were sorted on GFP expression and maintained in culture with cytidine before analysis. The black line corresponds to the isotype control, the red line to the anti-CTPS1 staining. **(B)** Same as (A) except that cytidine was removed or not from the cultures for up to 11 d. **(C)** Western blot of CTPS1 expression in cell lines obtained after sub-cloning of polyclonal cells shown in (A). Actin expression as a loading control. **(D)** CTPS activity measured in cell extracts of wild-type Jurkat cells and CTPS1-deficient Jurkat clones. Means with SEM of three independent experiments. Two-tailed unpaired *t* test; **P < 0.01. **(E)** Proliferation graphs from resazurin/resofurin assays of three CTPS1-KO Jurkat cell lines (#2, #3, and #5) and wild-type Jurkat cells in the presence or absence of cytidine or 3-deaza-uridine (3-DU) at the indicated concentrations. **(F, G, H)** Analysis of one of the CTPS1–KO cell lines (#5) and one control cell line for cell cycle progression (F), proliferation (G), and apoptosis (H) in the presence or absence of 200 µM cytidine or 40 µM 3-DU. **(F)** FACS dot-plots of cell cycle analysis showing incorporation of EdU and IP incorporation in control or CTPS1-KO cells. Diagram on the right showing the correspondence of the gates with the G1, G2, and S phases of the cell cycle. Lower graphs showing the percentages of cells in G1, G2, and S phases from FACS data. **(G)** Histograms from FACS analysis of CFSE staining dilution–based proliferation assay. **(H)** FACS dot-plots of expression of the apoptotic/cell death annexin V and 7-AAD at 72 h. Cells were seeded for 24 h, and then cultured in the presence or absence of 200 µM cytidine or 40 µM of 3-DU. **(B, C, D, E, F, G, H)** Jurkat correspond to Jurkat cells shown in panel (A) that have been transfected with an empty vector. **(A, B, E, F, G, H)** Data of one representative experiment of three independent experiments in (A), two in (B), three in (E) for Jurkat and Jurkat CTPS1-KO (#3), three in (G) and three in (H).

**Figure S4.**
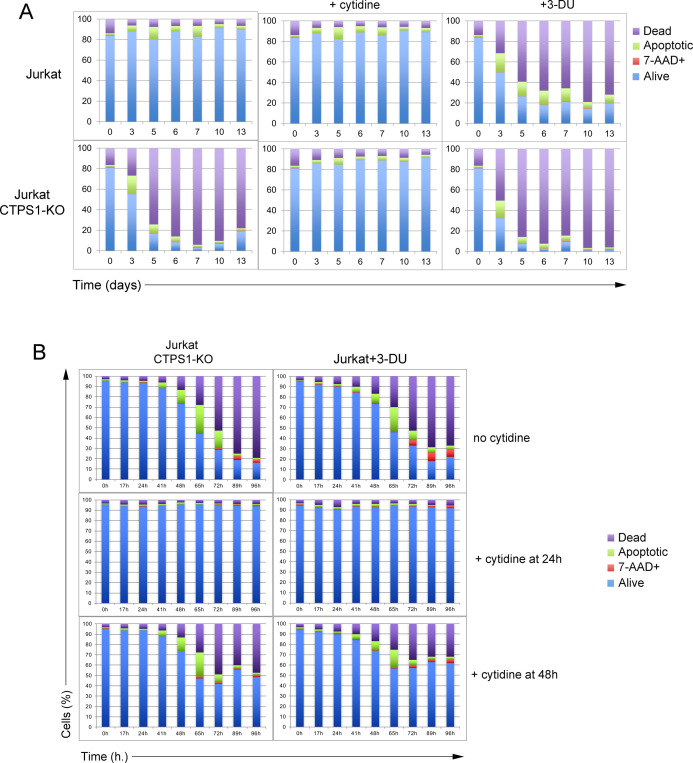
Inactivation of CTPS1 in Jurkat cells results in rapid cell death and can be counteracted by supplementation of cytidine at 24 h but not at 48 h. **(A, B)** Cells were seeded for 24 h, and then cultured in the presence or absence of 200 μM of cytidine or 40 μM of 3-deaza-uridine (3-DU). Apoptotic/cell death markers annexin V and 7-AAD were analysed by flow cytometry at day 3, 5, 6, 7, 10, and 13. Bar graphs from flow cytometry data as presented in Fig 4H showing the percentage (%) of dead, 7-AAD+, alive, and apoptotic cells at 17, 24, 41, 48, 65, 72, 89, and 96 h (h.). **(B)** Same as (A) except that 200 μM of cytidine has been added at 24 or 48 h. **(A, B)** Data of one representative experiment of three (A) or two (B) independent experiments.

**Figure 4.**
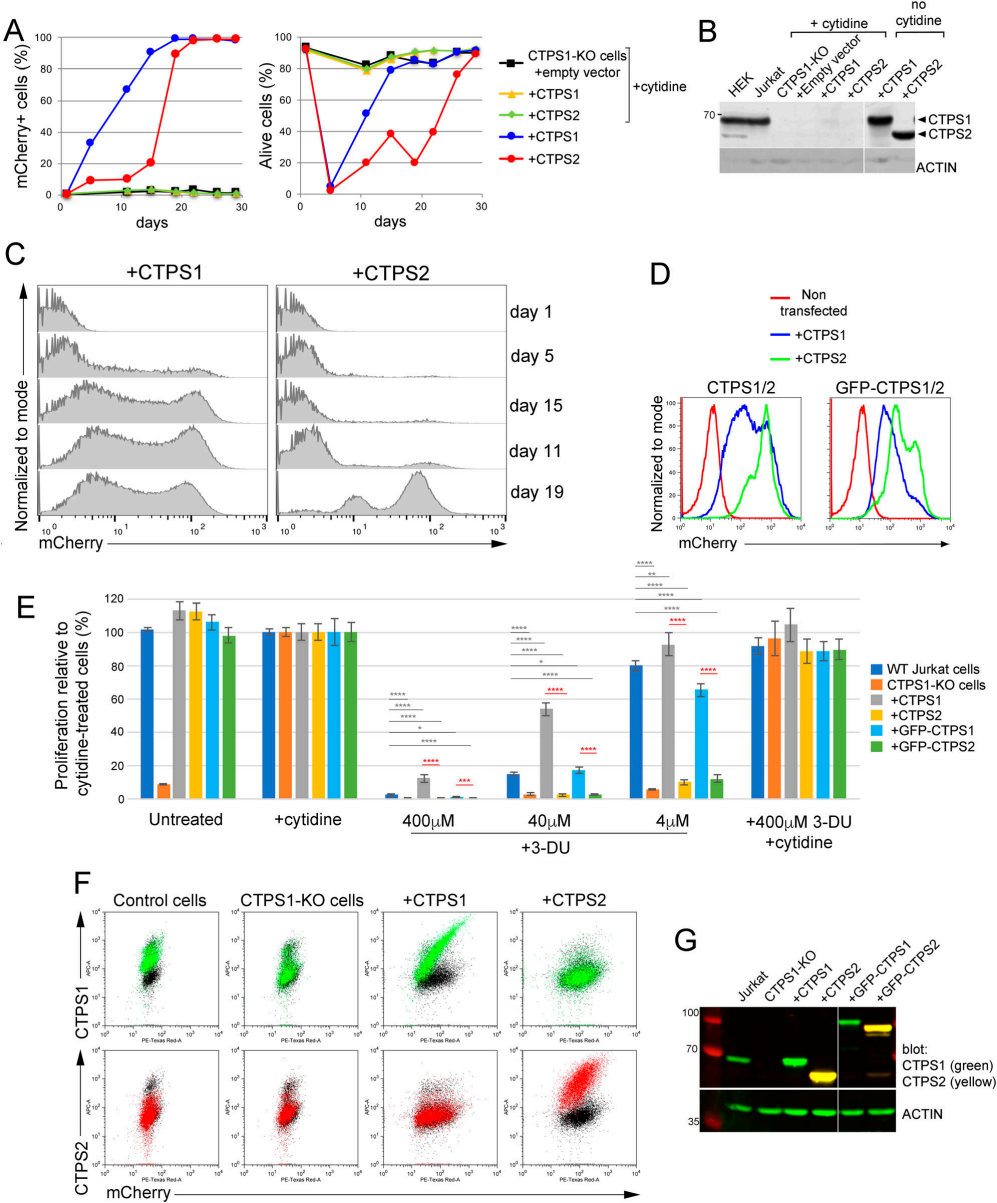
More CTPS2 than CTPS1 is required for proliferation of Jurkat cells. **(A, B, C)** CTPS1-deficient Jurkat cells infected with lentiviral expression vectors for CTPS1 or CTPS2 with mCherry as a reporter gene. Cells were then maintained in culture with or without cytidine. **(A)** Percentages (%) of mCherry-positive cells (left panel) and alive cells (right panel) based on FACS profiles. At day 0, 0.6%, and 0.46% of the cells infected with CTPS1 and CTPS2 were mCherry positive, respectively. **(B)** Western blots for CTPS1 and CTPS2 expression in Jurkat cell lysates at day 26. **(C)** Histogram profiles from FACS analyses of mCherry expression of cells during the culture without cytidine. **(D, E, F, G)** CTPS1-deficient Jurkat cells infected with high viral titers of lentiviral vectors for CTPS1 or CTPS2 expression with mCherry as a reporter gene allowing around 94% (CTPS1/CTPS2) and 70% (GFP–CTPS1/GFP–CTPS2) of mCherry-positive cells at day 0. Cells were then maintained for 17 d in culture without cytidine for selection. **(D)** Histogram profiles from FACS analyses of mCherry expression of cells at day 55. **(E)** Bar graphs of cell proliferation at day 5 from resazurin/resofurin assays of the different cell lines not treated (untreated) or in the presence or absence (untreated) of cytidine (200 µM) or with the indicated concentrations of 3-deaza-uridine (3-DU). Means with SEM of six experimental replicates from two independent experiments (three replicates each). Similar results at day 3 and 4. **(F)** Dot-plots from FACS analysis of intracellular CTPS1 (in green) or CTPS2 (in red) and mCherry reporter expression showing that the mCherry expression is proportional to CTPS1 or CTPS2 expression. Isotype in black. **(G)** Western blots for CTPS1 and CTPS2 expression (at day 37). **(B, G)** Actin expression as loading control. **(A, C, D, E)** Data of one representative experiment of three independent experiments in (A), three in (C), and three in (D). **(E)** Two-tailed unpaired *t* tests; n.s., no significance; *P < 0.05; **P < 0.01; ***P < 0.001; and ****P < 0.0001.

We previously reported the major role of *CTPS1* in the proliferation of activated T lymphocytes (28, 29). Multiple T lymphocyte cell lines express low levels or are negative for the expression of *CTPS2* at both RNA and protein levels, including the acute leukemia T-cell line Jurkat, in which *CTPS2* transcript and protein were undetectable (Figs 1 and 4B). We obtained Jurkat cells in which *CTPS1* was targeted by CRISPR–Cas9 (29). These cells were analysed for CTPS1 expression by intracellular cytometry staining. Most of them were negative for CTPS1, in contrast to cells that were targeted with a control guide, albeit a small fraction remained positive (∼20%) (Fig 3A). Cells were maintained in the presence of cytidine to avoid counterselection of the remaining cells expressing CTPS1. Removal of cytidine supplementation after a few days led to the rapid loss of the CTPS1-deficient cells to the benefit of CTPS1-positive cells that rapidly expanded in the culture (Fig 3B). The loss of CTPS1-deficient cells correlated with the appearance of a massive population of cells, whose shape in FSC/SSC representation likely corresponded to either dying or dead cells (data not shown) that was further confirmed by analysing CTPS1-KO clones (see below). This suggested that the CTPS1-expressing cells had a selective growth advantage in culture, whereas the CTPS1-deficient cells rapidly died. Because some cells still expressed CTPS1 after inactivation by CRISPR–Cas9, we sub-cloned these cells in the presence of cytidine to support their growth. Several clones were obtained in which CTPS1 expression was completely abolished (CTPS1-KO) (Fig 3C). These clones were further analysed for cell proliferation and CTPS activity. As expected, CTPS1-KO clones had no detectable CTPS activity (Fig 3D). When cytidine was removed, CTPS1-KO cells stopped proliferating (Fig 3E) and were arrested in the G1 cell cycle phase as early as 24 h (Fig 3F). In the presence of cytidine, CTPS1-KO cells had a comparable proliferation and cell cycle progression to that of control Jurkat cells (cultured with or without cytidine). Of note, similar to what was observed with wild-type HEK cells, cytidine supplementation had no impact on the proliferation of wild-type Jurkat cells indicating that CTP was not a limiting factor for their growth (Fig 3E). Further analysis of proliferation by CFSE incorporation and dilution confirmed that CTPS1-KO cells failed to proliferate in the absence of cytidine supplementation (Fig 3G). Inhibiting CTPS activity in wild-type Jurkat cells by 3-DU treatment led to a strong decrease in cell proliferation that was reversed by the addition of cytidine (Fig 3E and G). 3-DU treatment of Jurkat cells also resulted in a block in the G1 phase of the cell cycle (similar to that of CTPS1-KO cells in absence of cytidine) (Fig 3F). The effect of CTPS1 deficiency on cell death was further analysed in CTPS1-KO cells cultured in the presence of cytidine and then deprived of cytidine for 96 h (Figs 3H and S4A). Cytidine deprivation after 48 h led to the rapid accumulation of apoptotic (7AAD^−^ annexin V^+^) and dead (7AAD^+^ annexin V^+^) cells. Similar kinetics for the appearance of apoptotic and dead cells were observed when wild-type Jurkat cells were cultured in the presence of 3-DU. Apoptosis induced by cytidine starvation (for CTPS1-KO cells) or 3-DU treatment (for wild-type Jurkat cells) can be stopped or reduced by adding cytidine 24 or 48 h post-deprivation, respectively (Fig S4B). Taken together, these data demonstrate that CTPS1 is a key factor for Jurkat cell survival and proliferation.

### Differential roles of CTPS1 and CTPS2 in promoting proliferation

Our results regarding the respective role of CTPS1 and CTPS2 in the proliferation of HEK cells were in favor of a predominant role for CTPS1 in proliferation. To confirm this hypothesis, we compared the ability of CTPS2 or CTPS1 to restore the proliferation of Jurkat CTPS1–deficient cells. CTPS1-deficient cells were transduced with lentiviral vectors coding either for CTPS1 or CTPS2 along with a fluorescent mCherry reporter in the presence of cytidine and then grown in cytidine-free medium (Fig 4A, left panel). Transduction with a low viral titer in which less than 1% of cells were transduced allowed following the selective advantage of the transduced mCherry-positive population. From day 2, mCherry-positive cells transduced with CTPS1 rapidly expanded in culture, and all cells were mCherry positive at day 20. In contrast, expansion of mCherry-positive cells transduced with CTPS2 was delayed, and only at day 15 did mCherry-positive cells begin rapidly accumulated to reach 100% at day 23. In both cultures, selection of mCherry-positive cells was associated with an initial marked decrease in the global cell viability, caused by the cytidine starvation, that recovered by day 10 in correlation with the expansion of mCherry-positive cells in the culture (Fig 4A, right panel). As expected, expression of CTPS1 or CTPS2 was detectable in both cultures by Western blot and was barely observed in cultures that were maintained in the presence of cytidine in which mCherry-positive cells did not expand (Fig 4B). Interestingly, the mCherry staining revealed two main distinct populations in both CTPS1- and CTPS2-complemented cells (Fig 4C). The subpopulation with the highest level of mCherry decreased over time for the benefit of the population with the lowest level, suggesting a counterselection effect possibly due to toxicity associated with high expression of CTPS1 or CTPS2. However, the population of CTPS2-complemented cells with the lowest level which was selected over time expressed higher mCherry levels than the corresponding population in CTPS1-complemented cells (with the lowest mCherry expression).

We also observed higher levels of mCherry associated with CTPS2 complementation compared with CTPS1 when CTPS1-deficient Jurkat cells were complemented using high viral titers allowing up to 90% of infected cells after a few days of selection in the absence of cytidine (in contrast to the previous experiment) (Fig 4D). Similar findings were obtained when cells had been transduced with GFP-tagged forms of CTPS1 and CTPS2 (denoted as GFP–CTPS1 and GFP–CTPS2). Analysis of the proliferation of the different complemented Jurkat cell populations did not reveal any differences (Fig 4E). Furthermore, CTPS1-KO Jurkat cells complemented with CTPS2 forms were more sensitive to 3-DU treatment than cells complemented with CTPS1 forms, similar to our previous observations for CTPS1-deficient HEK cells that only expressed CTPS2 (CTPS1-KO cells, see Fig 2). Importantly, we showed that levels of mCherry expression directly correlated with levels of CTPS1 or CTPS2 expression analysed via intracellular staining in parallel to the mCherry expression (Fig 4F and G). Therefore, taken together, these data indicate that more CTPS2 than CTPS1 is required to maintain the proliferation of Jurkat cells.

**Figure 5.**
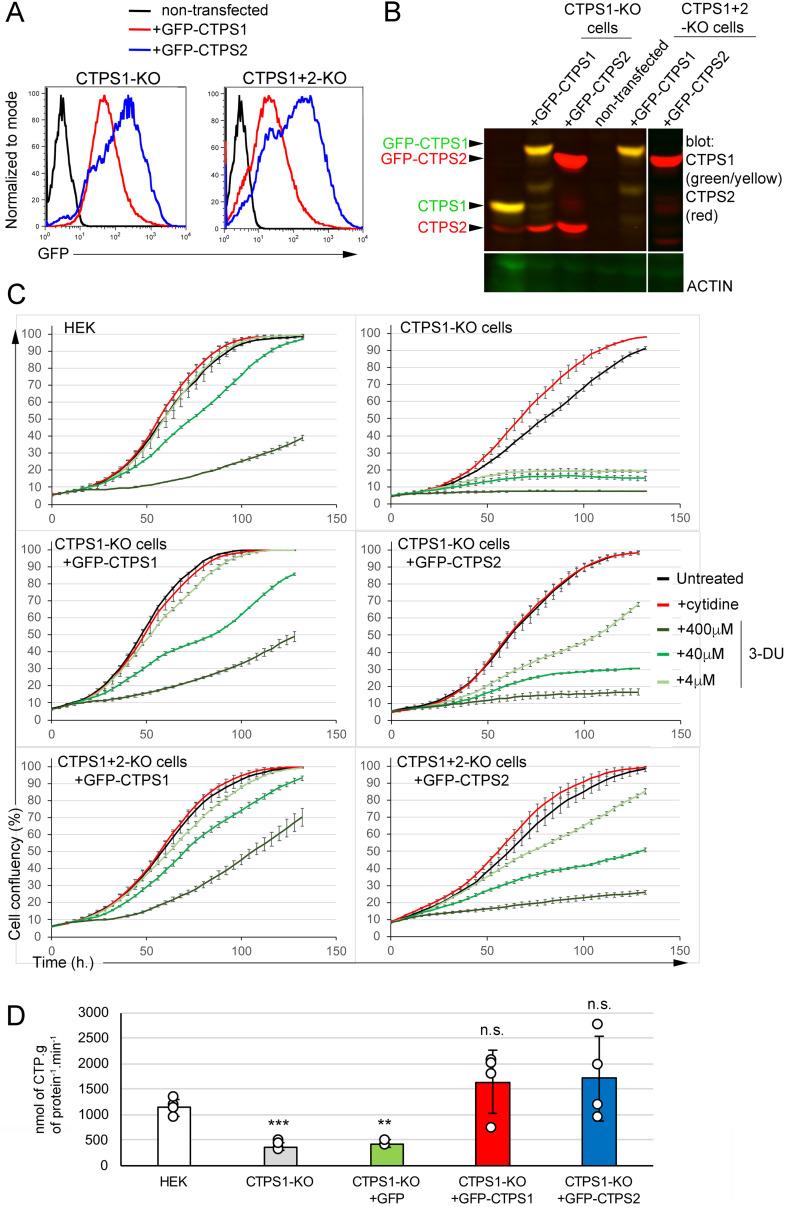
CTPS1 is more efficient than CTPS2 in restoring the proliferation of CTPS1-KO and CTPS1+2-KO HEK cells. **(A, B, C, D)** CTPS1-KO or CTPS1+2-KO cells were transfected with linearized C1 EGFP vectors containing GFP–CTPS1 or GFP–CTPS2. Cells were then maintained in culture without cytidine and sorted on GFP expression. **(A)** Histograms of GFP expression after sorting and culture in the absence of cytidine. **(B)** Western blots for CTPS1 and CTPS2 expression in cell lysates. Actin expression as a loading control. **(C)** Confluency curves as percentages (%) showing the proliferation. Confluency measurement using an IncuCyte Zoom system. Cells were seeded for 24 h, then untreated or maintained in the presence or absence of cytidine (200 µM) or 3-deaza-uridine with the indicated concentrations. **(D)** CTPS activity measured in cell extracts of CTPS1-KO cells reconstituted with GFP alone, GFP–CTPS1, or GFP–CTPS2. Means with SEM. Data from four independent experiments with replicates. Two-tailed unpaired *t* tests againt HEK values; n.s., no significance; **P < 0.01; ***P > 0.001. **(A, B, C)** Data of one representative experiment of three independent experiments in (A) and four in (C) except for CTPS1+2-KO cells with GFP–CTPS2 only tested two times.

**Figure S5.**
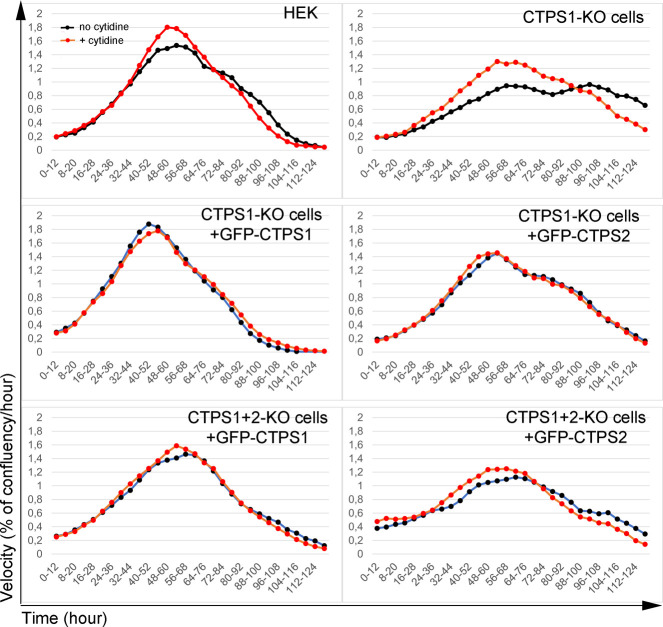
CTPS1 is more efficient than CTPS2 in restoring the proliferation of CTPS1-KO and CTPS1 + 2-KO HEK cells. Proliferation velocity, corresponding to Fig 5C. Velocity for each population in presence (red dotted line) or absence (black dotted line) of cytidine supplementation (200 μM), corresponding to the increase in confluency per hour on 12-h periods. Data of one representative experiment of four independent experiments except for CTPS1+2-KO cells with GFP–CTPS2 only tested two times.

Similar findings were obtained when CTPS1-deficient HEK cells (CTPS1-KO cells) and double CTPS1- and CTPS2-deficient HEK cells (CTPS1+2-KO cells) were complemented with GFP-tagged forms of CTPS1 or CTPS2 (Fig 5). GFP expression was higher in cells complemented with CTPS2 than that of CTPS1 (Fig 5A). Although cells with GFP–CTPS2 expressed high levels of CTPS2 (Fig 5A and B), their proliferation rate/velocity appeared to be reduced compared with GFP–CTPS1-expressing cells (Figs 5C and S5). As shown previously, cells complemented with CTPS2 forms were more sensitive to 3-DU treatment. Overall, the results from these two cell lines were convergent, and therefore, indicate that the role of CTPS2 is less important than that of CTPS1 to promote cell proliferation and that more CTPS2 is required to achieve the same rate of proliferation.

### Reduced enzymatic activity of recombinant CTPS2

**Figure S6.**
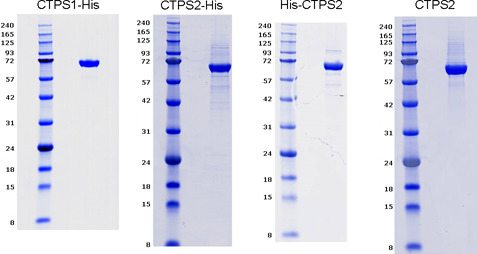
Purified recombinant CTPS1 and CTPS2 proteins. Analysis of purified recombinant tagged forms of CTPS2 and CTPS1 with C-terminal FLAG–histidine tag (CTPS1–His, CTPS2–His), CTPS2 with N-terminal histidine tag (His–CTPS2) and CTPS2 without a tag (CTPS2) used in Fig 6 by SDS–PAGE with 10% acrylamide gels. Molecular weight markers on the right. Arrows indicate the purified proteins. The purity is >90% for each protein.

**Figure 6.**
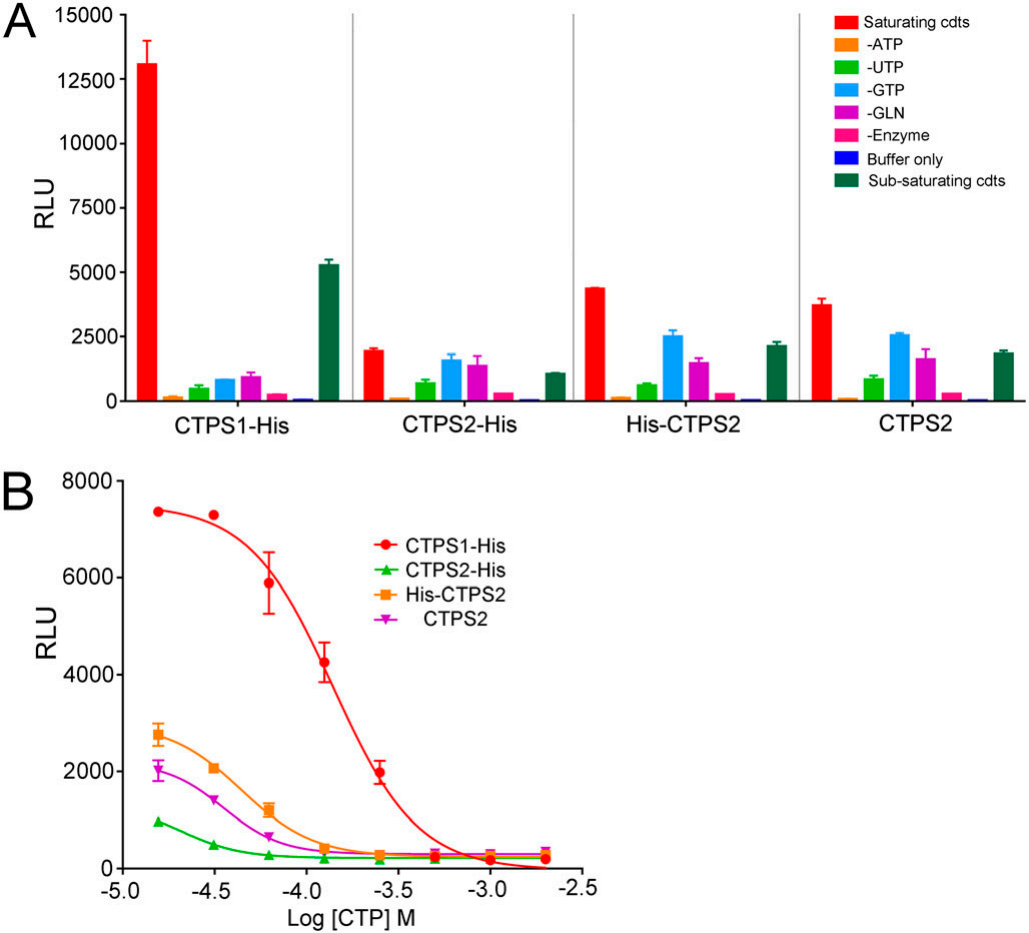
In vitro enzymatic activity of recombinant CTPS2 is lower than that of CTPS1. **(A, B)** Enzymatic activity of purified recombinant tagged forms of CTPS2 and CTPS1 with C-terminal histidine tag (CTPS1–His and CTPS2–His), CTPS2 with N-terminal histidine tag (His–CTPS2) and CTPS2 without a tag, analysed using ADP-Glo kinase assays (A) in the presence of buffer only (buffer only), all substrates in saturating (saturating cdts; cdts, conditions) or sub-saturating concentrations (sub-saturating cdts), under conditions in which one of the substrates was removed (-ATP, -UTP, -GTP, -GLN) or without enzyme (-enzyme) with all substrates under saturating conditions (B) with all substrates in saturating concentrations and with increasing concentrations of CTP. **(A, B)** Data of one representative experiment of two independent experiments with triplicates. Means with SEM of experimental triplicates. RLU, relative luminescence units.

The reduced ability of CTPS2 to sustain proliferation could be explained by lower enzymatic activity and/or differences in the regulation of its enzymatic activity as suggested by recent observations (21, 33). To test this possibility, CTPS activity was first examined in GFP–CTPS1- or GFP–CTPS2-reconstituted HEK cells and found to be roughly similar (Fig 5D). This likely indicates that indeed CTPS2 enzymatic activity is weaker than that of CTPS1 as GFP–CTPS2-reconstituted cells expressed higher levels of GFP–CTPS2 (compared with GFP–CTPS1-expressing cells). To confirm this observation, the enzymatic activity of recombinant purified human CTPS2 and CTPS1 proteins produced in HEK cells was tested in vitro (Fig S6). CTPS1 and CTPS2 were produced and purified using C- and N-terminal tags. All CTPS2 forms exhibited reduced enzymatic activity when compared with CTPS1 (Fig 6A). Interestingly, CTP was more potent at inhibiting CTPS2 than CTPS1 (Fig 6B). These observations are in line with the recent observations, indicating that CTPS2 contains two sites of negative feedback regulation by CTP in contrast to CTPS1 that only has one (33). Hence, the weaker intrinsic enzymatic activity of CTPS2 likely accounts for the diminished ability of CTPS2 to support cell proliferation.

### CTPS1 is essential for cancer cell growth in most cancer cell lines

**Figure 7.**
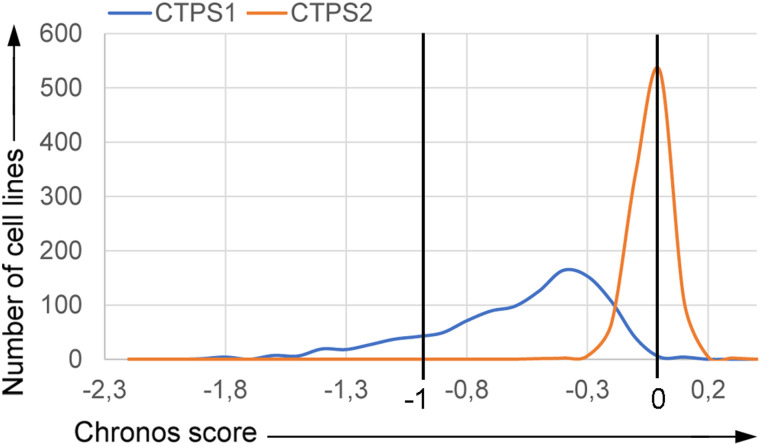
CTPS1 is an essential gene for survival and proliferation of most cancer cell lines. Chronos score of 1,064 cell lines for CTPS1 and CTPS2, extracted from the DepMap 22Q1 Public + Score Chronos dataset. Counterselection of CRISPR-targeted cells leads to guide depletion and is translated by a negative score. A score of 0 corresponds to a non-essential gene, whereas a negative score shows a dependency for the concerned gene. The score of −1 corresponds to the median of all essential genes from the DepMap database. For this DepMap release: (44).

To extend our observations on tumor cell lines other than HEK and Jurkat cell lines, we further examined the requirement for CTPS1 and CTPS2 for cell growth and survival of a large number of tumor cell lines from different tissues using data from the Project Achilles CRISPR-based genome-scale loss-of-function screening (34, 35 *Preprint*, 36 *Preprint*, 37). Dependency scores for *CTPS1* and *CTPS2* were extracted through the DepMap website. Interestingly, although the *CTPS2* Chronos guide depletion scores were close to 0 (mean = −0.038), highlighting its non-essentiality in cancer cell survival and proliferation, we observed a CTPS1 guide depletion (high negative scores towards −1) in most of the 1,070 cell lines (Chronos score mean = −0.579), indicative of its essentiality (Fig 7). Based on these analyses, 2 out of 1,064 cell lines (CTPS2) and 662 out of 1,070 cell lines (CTPS1) were considered to be dependent for their viability of CTPS1 or CTPS2, respectively. Taken together, these results extended our observations showing that most of the cancer cell lines tested (>1,000 different cell lines) are highly dependent on CTPS1 but not or less on CTPS2 for their survival and proliferation.

## Discussion

Our results in HEK and Jurkat cell line models indicate that CTPS1 and CTPS2 are partially redundant for cell proliferation. The role of CTPS2 in proliferation is less important than that of CTPS1. Our results indicate that higher levels of CTPS2 than CTPS1 are necessary to achieve similar proliferation rates. This could be explained by the lower intrinsic enzymatic activity of CTPS2, although we cannot formally exclude other mechanisms independent of the intrinsic enzymatic activity. Along these lines, we previously showed that CTPS1 was critical to sustain proliferation of activated T lymphocytes, despite their expression of CTPS2. Thus, in activated T lymphocytes, CTPS2 does not compensate for the defect in CTPS1, indicating that the role of CTPS2 to promote proliferation is minimal. Furthermore, in support of differential roles of CTPS1 and CTPS2, recent studies showed that CTPS2 enzymatic regulation is different compared with CTPS1. In contrast to CTPS1, CTPS2 contains two sites of negative feedback regulation by CTP that might contribute to its lower activity by being more sensitive to CTP and thus more rapidly inhibited than CTPS1 (33). Our results also indicate that CTPS2 is more prone to inhibition by 3-DU, a competitive inhibitor of UTP that very likely reflects a low affinity to UTP, thus leading to a lower intrinsic enzymatic activity (compared with CTPS1). Consequently, both specific regulatory and intrinsic determinants account for the low activity of CTPS2. Altogether, the higher activity of CTPS1 may be required in vivo to rapidly meet a critical need of CTP in highly proliferating cells, like activated T lymphocytes or cancer cells, whereas CTPS2, which has a lower activity, may be rather involved in maintaining basal cellular CTP pools in general.

Interestingly, full depletion of the CTPS activity (by inactivation of CTPS1 and CTPS2) has distinct consequences in Jurkat versus HEK cells, although proliferation is completely abrogated in both cell lines following CTPS inactivation. Jurkat cells without CTPS1 are blocked in the G1 phase of the cell cycle (if no CTP is provided to compensate for the lack of CTPS activity) and rapidly die by apoptosis within the next 48 h. In contrast, HEK cells deficient for both CTPS1 and CTPS2 stopped growing in the absence of cytidine supplementation, but only a small fraction of cells died even after 10 d of CTP deprivation. Although these cells are stopped in their proliferation, a large proportion still remained stuck in the S phase of the cell cycle, but Edu staining was low, suggesting an abnormal “low activity” S phase. When CTP was added back into the medium, cells rapidly recovered and proliferated even after 14 d of deprivation. These results indicate that HEK cells in the absence of CTPS activity or CTP are resistant to apoptosis and become quiescent (defined as a reversible non-proliferating state). Such behavior might be explained by the embryonic origin of HEK cells and by their capacity to survive under various selective conditions (38). Deprivation of cellular CTP likely results in stalled replication forks, leading to replicative stress, a complex phenomenon that occurs when replication is impeded (39). Depending on the nature of the replicative defect or the source of the replicative stress, the response can involve different mechanisms and consequences including apoptosis. However, the effect of CTP deprivation or defective CTPS activity on replication stress response is currently unknown and warrants further study. We also noticed that the size of HEK cells deficient for both CTPS1 and CTPS2 markedly increased upon CTP deprivation. Jurkat cells treated with 3-DU, CTPS1-deficient Jurkat cells, and CTPS1-deficient T cells of patients also exhibited a larger size than that of their normal size (Minet N., Martin E. and S. Latour, unpublished observations). We have no clear explanation for this phenomenon.

Our results are important in the context of the development of CTPS1 inhibitors. We previously proposed that CTPS1 could represent a therapeutic target to suppress adaptive immune responses by specifically blocking the expansion of T lymphocytes and specific inhibitors of CTPS1 have been recently obtained that block the proliferation of Jurkat cells and human primary T cells (33). Because high levels of CTPS activity have been reported in cancer cells (22, 23), in particular in lymphomas (24), inhibition of CTPS1 represents a potent therapeutic approach to block cancer progression. Our analysis of a large number of tumour cell lines (>1,000) from the Project Achilles CRISPR-based genome-scale loss-of-function screening, and gene copy numbers from CCLE data confirm that *CTPS1* is an essential factor in cell growth for most of these cell lines. Furthermore, our experimental results with HEK and Jurkat cell lines suggest that in tumor cells expressing CTPS1 only or in association with a low expression of CTPS2 (like T cell lines), inhibition of CTPS1 could lead to the rapid death of the cells by apoptosis. However, in cells co-expressing both CTPS1 and CTPS2, inhibition of CTPS1 would be not sufficient to block proliferation as CTPS1-deficient HEK cells can still proliferate (although the rate of proliferation was diminished). The contribution of CTPS2 to proliferation thus appears to be essential when CTPS1 is absent, whereas minimal when CTPS1 is present. Thus, the efficacy of a treatment targeting CTPS1 might be particularly dependent on the level of CTPS2 in cancer cells.

Interestingly, among the different tumor cell lines of hematopoietic origin we tested, T-cell lymphomas appear to be those in which the expression of CTPS2 is the lowest or undetectable. The myeloid cell line U937 also shows a weak expression of CTPS2. In contrast, B-cell lymphomas expressed more substantial levels of CTPS2, and a recent study suggested that the inactivation of both CTPS1 and CTPS2 is required to fully block Epstein Barr virus–driven B-cell proliferation (40). Thus, neoplasms of T-cell origin, for which there is an important unmet medical need may represent a primary indication for CTPS1 inhibition.

Finally, as we previously observed for CTPS1-deficient primary T cells (14, 15), supplementation of CTPS-deficient HEK and Jurkat cells with cytidine through the salvage pathway restores proliferation. Hence, delivery of cytidine (or of a CTP precursor) could mitigate unwanted CTP deprivation–induced cell death or immunosuppression (in the case of cancer treatment) that might result from the use of therapeutic CTPS inhibitors.

In conclusion, our study is an important contribution to the understanding of the respective roles of CTPS1 and CTPS2 in cell proliferation, in particular in cancer cells, for which limited information is currently available.

## Materials and Methods

### Plasmids

Single-guide RNAs (sgRNAs) targeting exons 6 or 10 of *CTPS1* or exons 5 and 10 of *CTPS2* were designed as previously described (41) and cloned in the lentiCRISPR V1 (pXPR_001) (Addgene plasmid 49535) or pSpCas9(BB)-2A-GFP (PX458) (Addgene plasmid 48138) vectors. The sgRNA sequences were as follows: CTPS1 exon 6 F: CACCGAGTGTTCGGGAACTTAG/R: AAACCTAAGTTCCCGAACACTC and 10 F: CACCGGCTTCGTGGTAGCGCAC/R: AAACGTGCGCTACCACGAAGCC; CTPS2 exon 5 F: CACCGCGAAGGAATGCCGTTTG/R: AAACCAAACGGCATTCCTTCG and 10 F: CACCGGAAGATCACTGAAACCG/R AAACCGGTTTCAGTGATCTTC. Extinction efficiency was verified by Western blot or intracellular staining of puromycin-selected or GFP-sorted cell bulks before sub-cloning. For complementation experiments, full-length CTPS1 and CTPS2 cDNAs were obtained by PCR as previously described (28) using the Q5 High-Fidelity DNA Polymerase (New England Biolabs). For CTPS1, forward 5′-AAGCAGACTAGTCCACCATGAAGTACATTCTGG-3′ and reverse 5′-AAGCAGGCGGCCGCTCAGTCATGATTTATTGATGGAAACTTC-3′ were used. For CTPS2, forward 5′-AAGCAGACTAGTCCACCATGAAGTACATCCTG-3′ and reverse 5′-AAGCAGGCGGCCGCTCAGCTTATTTCCAACTCAGC-3′ were used. The cDNAs were verified by sequencing and inserted into a bicistronic lentiviral expression vector encoding mCherry as a reporter (pLVX-EF1α-IRES-mCherry Vector, cat#631987; Clontech). For GFP tagging, the cDNAs were inserted into a pEGFP-C1 vector (cat#6084-1; Clontech) using the following primers. For CTPS1, forward 5′-AAGCAGGGTACCCCACCATGAAGTACATTCTGG-3′ and reverse 5′-AAGCAGGGATCCTCAGTCATGATTTATTGATGGAAACTTC-3′; for CTPS2, forward 5′-AAGCAGGGTACCCCACCATGAAGTACATCCTG-3′ and reverse 5′-AAGCAGGGATCCTCAGCTTATTTCCAACTCAGC-3′ were used. All constructs were validated by Sanger sequencing using the BigDye Terminator v3.1 Cycle Sequencing Kit (Life Technologies) and a 3500xL Genetic Analyzer (Applied Biosystems) according to the manufacturer’s instructions. Sequence analysis was performed using DNADynamo (BlueTractorSoftware).

### CTPS1 and CTPS2 gene expression analysis

Total RNA was isolated from cell lines using the RNeasy Mini kit (QIAGEN) and reverse transcription was performed using Superscript II First Strand Synthesis System (Invitrogen). cDNAs were used as a template for CTPS1 and CTPS2 gene expression by qRT-PCR. Gene expression assays were performed with Assayson-Demand probe and primer combinations (CTPS1, Hs01041858; CTPS2, Hs00219845; GAPDH, Hs027558991) from Applied Biosystem labelled with 6-carboxy-fluorescein (FAM) dye and universal reaction mixture. Real-time quantitative PCRs for GAPDH, CTPS1, and CTPS2 were performed in triplicate using a LightCycler VIIA7 System (Roche). Expression levels were determined by relative quantification using the comparative threshold cycle method 2DDCt in which DDCt is determined as follows: (Ct^target gene^ − Ct^reference gene^) target tissue – (Ct^target gene^ − Ct^reference gene^) calibrator tissue. The results shown in arbitrary units (a.u.) have been normalized to GAPDH gene expression (reference gene) and are presented as the relative change in gene expression normalized against the calibrator sample corresponding to HEK cells.

### Cell culture

Jurkat cells (RRID:CVCL_0065; ATCC) were cultured in RPMI, 10% FBS, 1% penicillin–streptomycin (PS) (complete RPMI). HEK 293T cells (RRID:CVCL_0063; ATCC) were cultured in DMEM, 10% FBS, 1% PS (complete DMEM). Calcium and magnesium-free PBS and 1x trypsin–EDTA were, respectively, used to wash the cells and to detach adherent cells. All of the abovementioned reagents were from Life Technologies. CTPS1- or/and CTPS2-deficient cell lines were maintained with 200 µM of cytidine (Sigma-Aldrich). 3-Deaza-uridine (3-DU) (Sigma-Aldrich) was used as a non-selective inhibitor of CTPS1 and CTPS2.

### Cell transfection and transduction

HEK 293T cells were transfected by electroporation using the Gene Pulser Xcell (Bio-Rad) or by lipofection with Lipofectamine 2000 (Life Technologies). pEGFP-C1 plasmids with CTPS1 or CTPS2 were linearized using the ApaLI restriction enzyme (New England Biolabs) before electroporation, purified on High Pure PCR Product Purification Kit columns (Roche), and eluted in water. After electroporation, cells having incorporated the vector were selected using puromycin (InvivoGen) for the pXPR_001 vector, sorted based on GFP expression for the PX458 vector and the pEGFP-C1 vector, or on mCherry expression for the pLVX vector (RRID:Addgene_174088) using cell sorter (SH800; Sony). For low efficiency transductions, Jurkat CTPS1-KO cells were infected lentiviral particles containing lentiviral vectors for CTPS1 or CTPS2 expression as previously described (29). For high efficiency transductions, viral particles produced at the VVTG platform at Hospital Necker (VVTG platform; SFR Necker) were concentrated by ultracentrifugation, and the cells were infected with a MOI of 10.

For CRISPR–Cas9-mediated gene inactivation, Jurkat cells were transfected by electroporation using the Nepa21 electroporator (Nepagene) with PX458 plasmids, sorted on EGFP expression, and sub-cloned as previously described (29). HEK 293T cells were electroporated with lentiCRISPR V1 plasmids, selected using puromycin, and sub-cloned. HEK cells in which both CTPS1 and CTPS2 were inactivated were obtained by inactivation of CTPS2 using the same approach on one of the CTPS1-deficient HEK cell lines obtained (CTPS1-KO clone#5). Cells were maintained with 200 µM of cytidine after electroporation to avoid counterselection.

### Cell proliferation

For the resazurin proliferation assay, cells were seeded at a density of 150,000 cells/ml in 96-well plates. At 24 and 48 h, 10 µl of CellTiter-Blue (Promega) was added, and cells were further incubated at 37°C for up to 4 h. Absorbance at 560/595 nm was measured and analysed according to the manufacturer’s instructions using a Tecan Infinite 200 Pro plate reader (Tecan Life Sciences). For CSFE incorporation–based proliferation assay, cells were incubated overnight with 3 mM of hydroxyurea (Sigma-Aldrich), washed, and labelled with CFSE (Invitrogen) according to the manufacturer’s instructions, resuspended in complete medium and cultured for 4 d. Cells were then analysed using an LSRFortessa X-20 cytometer and FlowJo software (BD Biosciences). Proliferation of HEK cells was analysed as a percentage of confluency. For that, cells were seeded in complete culture medium at a density of 6,250 cells/cm^2^ for 24 h and placed in an IncuCyte Zoom Live-Cell analysis system (Sartorius). Phase contrast and GFP fluorescence pictures were taken every 3 h and analysed using a confluency mask.

### Cell cycle

Cells were washed, resuspended in complete medium, and cultured for 24 h with the indicated concentrations of cytidine or 3-DU. The cells were then incubated for a further 1 h with 10 µM EdU, fixed, and stained according to the manufacturer’s instructions (Click-iT EdU Cell Proliferation Kit for Imaging; Life Technologies). All data were collected on LSRFortessa X-20 cytometer, and data were analysed using FlowJo software (RRID:SCR_008520; BD Biosciences).

### Annexin V/7-AAD apoptosis

Cells were washed and seeded at a density of 150,000 cells/ml in the presence or absence of 40 µM of 3-deaza-uridine or 200 µM of cytidine in 24 well plates (one well per time point). At each time point, the cells were stained with FITC-conjugated annexin V and 7-AAD according to the manufacturer’s instructions (BD Biosciences) and analysed using a LSRFortessa X-20 cytometer and FlowJo software (RRID:SCR_008520; BD Biosciences). HEK cells, maintained with 200 μM of cytidine, were washed, detached using trypsin–EDTA (Life Technologies), and seeded in six-well plates at a density of 26,000 cells/cm^2^ in the presence or absence of 200 μM of cytidine in DMEM, 10% FBS, 1% penicillin–streptomycin. At each time point, the cells were imaged using an Axio Vert A1 microscope (Zeiss) at the indicated magnification. The imaged cells were then detached using trypsin, mixed with their supernatant, and stained as described for the Jurkat cells. With the exception of the CTPS1+2-KO cells that never reached confluency, all the other cell populations were passaged (seeding of 26,000 cells/cm^2^) in parallel when they approached or reached confluency and maintained under the same culture conditions.

### Intracellular FACS

The cells were fixed and permeabilized using the IntraPrep Permeabilization Reagent (A07803; Beckman Coulter) according to the manufacturer’s instructions. Cells were stained first with an anti-CTPS1 antibody (ab133743; Abcam), an anti-CTPS2 antibody (ab190462; Abcam), or an isotype-matched antibody (rabbit IgG, ab172730; Abcam) and then labeled with an AF647-goat anti-rabbit secondary antibody. All data were collected on an LSRFortessa cytometer and analysed using FlowJo version 9.3.2 software (RRID:SCR_008520; BD Biosciences).

### Western blot

Cell lysates were prepared and analysed using standard procedures as previously described (28, 29). The following antibodies were used for immunoblotting: rabbit polyclonal anti-actin (cat# A2066; Sigma-Aldrich), rabbit monoclonal anti-CTPS1 (ab133743; Abcam), rabbit polyclonal anti-CTPS2 (ab190462; Abcam), rabbit anti-GFP (2956S; Cell Signaling). The following antibodies were used for detection: IRDye 680RD or IRDye 800CW–conjugated goat anti-rabbit (Li-cor 925-68071, Li-cor 925-32211) or goat anti-mouse (Li-cor 925-68070 and Li-cor 925-32210) and membranes analysed with the Odyssey CLX imager (Li-Cor).

### CTPS activity measurement

CTPS activity was measured in cell extracts as previously described (28, 29, 42). Briefly, cell pellets were sonicated on ice. Thirty micrograms of proteins were used for measuring CTPS activity in reaction mixture containing 1.7 mM EDTA, 13 mM MgCl2, 1.3 mM ATP, 0.2 mM GTP, 13 mM glutamine, 1.3 mM phosphoenolpyruvate, 10 mM NaF, 1.3 mM UTP, and 10 μM stable CTP isotope as an internal standard in 10 μl Hepes buffer, 70 mM, at pH 8.0. The reaction mixtures were incubated at 37°C for 90 min, and then the enzymatic reaction was stopped by the addition of 2 vol of HClO4. 5 μl of sample were injected onto an Acquity HSS T3 column, 1.8 μm particle size, 2.1 Å∼ 100 mm (Waters), connected to an Acquity H-Class ultra-performance liquid chromatography interfaced with a Xevo TQ-S triple-quadrupole mass spectrometer (Waters), both controlled by MassLynx software (MassLynx, RRID:SCR_014271; Waters). The CTP identification and detection were performed in the electrospray positive ion mode with multiple reaction monitoring mode (MRM). Quantification was performed using TargetLynx software. The threshold of CTPS activity detection corresponds to the mean of activity detected in Jurkat cells deficient for CTPS1 (that did not express CTPS2) from four independent experiments (51 ± 5 nmol of CTP/g protein/min) (27).

### CTPS activity on purified enzymes

The purified N-terminal His_6_-tag with extended linker form containing a cleavage site for the TEV protease (underlined) CTPS2 form (MDHHHHHHDTTENLYFQGGSGS-CTPS1), and the C-terminal FLAG–His_8_-tag CTPS1 and CTPS2 forms (CTPS1/CTPS2-GGDYKDDDDKGGHHHHHHHH) were provided by Proteros. Briefly, tagged proteins were produced in HEK cells and purified by Ni affinity chromatography. CTPS2 without tag was obtained by digestion of the His–CTPS2 with the TEV protease and purified by size exclusion chromatography. Purity of the proteins was >90% after verification by peptide finger mass print analysis and by analysing 5 μg of proteins by SDS–PAGE on 10% acrylamide gels revealed by Coomassie blue staining (as shown in Fig S6). CTPS activity of purified CTPS1 and CTPS2 proteins was then analysed using ADP-Glo Kinase assay (Promega). CTPS1 and CTPS2 were diluted in assay buffer (50 mM Trizma, 10 mM MgCl_2_, 2 mM L-cysteine, 0.01% Tween-20, pH 8.0) and enzyme reaction initiated by addition of substrates and co-factors at sub-saturating concentrations (0.31 mM UltraPure ATP, 0.034 mM GTP, 0.48 mM UTP, 0.19 mM L-glutamine for CTPS1 or 0.35 mM UltraPure ATP, 0.025 mM GTP, 0.5 mM UTP, 0.06 mM L-glutamine for CTPS2; final enzyme concentration 0.8 μM). Sub-saturating concentrations were determined per isoform by performing individual substrate titrations against a mastermix of remaining key substrates and co-factors held at the same saturating concentrations for each isoform (2 mM UltraPure ATP, 0.1 mM GTP, 2 mM UTP, 2 mM L-glutamine) (43). Of note, the different activity assay conditions which vary slightly in reactant concentrations for CTPS1 and CTPS2 were determined across multiple experiments. After incubation for 50 min at 20°C (within the linear phase of reaction) the reaction was terminated, and ADP product formation quantified using the ADP-Glo Max assay system (Promega) according to the manufacturer’s instructions. Michaelis–Menten plots were subsequently determined per substrate and co-factor over multiple repeat experiments to derive final activity assay concentrations.

### Analysis of the requirement for CTPS1 and CTPS2 in cancer cells

The dependency scores for CTPS1 and CTPS2 from the Project Achilles CRISPR-based genome-scale loss-of-function screening were downloaded through the Cancer Dependency Map website (https://depmap.org/) using the latest available data (DepMap 22Q1 Public + Score Chronos dataset).

## Data Availability

Datasets and reagents generated during the current study are available from the corresponding author on reasonable request. DepMap 22Q1 public data are available on (44).

## Supplementary Material

Reviewer comments
